# Synergistic detoxification of aflatoxin B1 using paraprobiotics and phytochemical extracts: quantitative analysis and mechanistic insights

**DOI:** 10.3205/dgkh000650

**Published:** 2026-06-02

**Authors:** Omid Haji Kandi, Seyed Mohammad Mahdi Hamdi, Mansour Bayat, Maryam Tajabadi Ebrahimi

**Affiliations:** 1 Department of Biology, CT.C, Islamic Azad University Tehran, Iran; 2 Department of Veterinary Pathobiology, SR.C., Islamic Azad University, Tehran, Iran

**Keywords:** aflatoxin B1, paraprobiotics, Lactobacillus brevis, Lactobacillus paracasei, Punica granatum, Asparagus khorasanensis, HPLC, detoxification, synergy

## Abstract

**Background:**

Aflatoxin B1 (AFB1), a potent group I carcinogen produced by Aspergillus species, poses a significant food safety challenge. This study evaluates the synergistic efficacy of heat-inactivated *Lactobacillus* *(L.) brevis* and *L. paracasei *(paraprobiotics) combined with ethanolic extracts of *Punica (P.) granatum* and *Asparagus (A.) khorasanensis* for AFB1 detoxification, extending prior findings on their anti-aflatoxigenic properties via gene expression modulation.

**Methods:**

AFB1 was produced using *Aspergillus flavus* PTCC 5006. Paraprobiotics were prepared by thermal inactivation, with cell wall integrity assessed via scanning electron microscopy (SEM) and Fourier-transform infrared spectroscopy (FTIR). Phytochemicals were characterized using high-performance liquid chromatography with diode-array detection (HPLC-DAD). AFB1 levels were quantified via HPLC with fluorescence detection (limit of detection [LOD]: 0.25 µg/mL; limit of quantification [LOQ]: 0.75 µg/mL). Dose-response relationships and synergy were evaluated using the Chou-Talalay combination index (CI).

**Results:**

Paraprobiotics reduced AFB1 by 47.2% (95% CI: 44.8–49.6%, Cohen’s d=1.8) at 10^8^ CFU/mL, outperforming viable probiotics (30.6%, p<0.001). The binary extract mixture (250 µg/mL each) achieved a 43.7% reduction (95% CI: 40.5–46.9%, CI=0.78). The combined treatment yielded an 85.8% reduction (95% CI: 82.3–89.3%, CI=0.65, p<0.001), indicating strong synergy, correlated with downregulated aflatoxin biosynthesis genes (*aflD, aflT, aflR, aflM*).

**Conclusion:**

The paraprobiotic-phytochemical combination offers a dual-mechanism approach for AFB1 detoxification, integrating physical sequestration and transcriptional suppression. This strategy holds promise for food safety applications, warranting further in vivo and food matrix studies.

## Zusammenfassung

**Hintergrund:**

Aflatoxin B1 (AFB1), ein potentes Karzinogen der Gruppe I, das von Aspergillus-Arten produziert wird, stellt eine erhebliche Herausforderung für die Lebensmittelsicherheit dar. Diese Studie evaluiert die synergistische Wirksamkeit von hitzeinaktiviertem *Lactobacillus brevis* und *L. paracasei* (Paraprobiotika) in Kombination mit ethanolischen Extrakten von *Punica granatum* und *Asparagus khorasanensis* zur Entgiftung von AFB1 und erweitert damit frühere Erkenntnisse über ihre anti-aflatoxigenen Eigenschaften via Modulation der Genexpression.

**Methode:**

AFB1 wurde mittels *Aspergillus flavus* PTCC 5006 hergestellt. Paraprobiotika wurden durch thermische Inaktivierung präpariert; die Zellwandintegrität wurde mittels Rasterelektronenmikroskopie (REM) und Fourier-Transformations-Infrarotspektroskopie (FTIR) untersucht. Phytochemikalien wurden mittels Hochleistungsflüssigkeitschromatographie mit Diodenarray-Detektion (HPLC-DAD) charakterisiert. Die AFB1-Konzentrationen wurden mittels HPLC mit Fluoreszenzdetektion quantifiziert (Nachweisgrenze [LOD]: 0,25 µg/mL; Bestimmungsgrenze [LOQ]: 0,75 µg/mL). Dosis-Wirkungs-Beziehungen und Synergismus wurden unter Verwendung des Kombinationsindex (CI) nach Chou-Talalay evaluiert.

**Ergebnisse:**

Paraprobiotika reduzierten AFB1 um 47,2% (95%-KI: 44,8–49,6%, Cohen’s d=1,8) bei 10^8^ KBE/mL und übertrafen damit lebende Probiotika (30,6%, p<0,001). Die binäre Extraktmischung (je 250 µg/mL) erzielte eine Reduktion um 43,7% (95%-KI: 40,5–46,9%, CI=0,78). Die Kombinationsbehandlung führte zu einer Reduktion um 85,8% (95%-KI: 82,3–89,3%, CI=0,65, p<0,001), was auf einen starken Synergismus hinweist. Dieser korrelierte mit der Herunterregulation der Aflatoxin-Biosynthesegene (*aflD, aflT, aflR, aflM*).

**Schlussfolgerung:**

Die Paraprobiotika-Phytochemikalien-Kombination bietet einen dual-mechanistischen Ansatz zur AFB1-Entgiftung, der physikalische Sequestrierung und transkriptionelle Suppression integriert. Diese Strategie zeigt vielversprechendes Potenzial für Anwendungen in der Lebensmittelsicherheit und erfordert weitere Studien in vivo und in Lebensmittelmatrices.

## Introduction

Aflatoxin B1 (AFB1), a mycotoxin produced by *Aspergillus (A.) flavus* and* A. parasiticus*, is classified as a group I carcinogen by the International Agency for Research on Cancer, with established links to hepatocellular carcinoma, particularly in regions with high contamination and hepatitis B prevalence [1], [2]. Contaminating approximately 25% of global agricultural commodities, including cereals, nuts, and dairy products, AFB1 poses a significant threat to food safety and public health [3], [4]. Conventional detoxification methods, such as physical removal or chemical treatments, are limited by cost, efficacy, or secondary contamination risks [5], [6]. Biological approaches using lactic acid bacteria (LAB) have demonstrated potential for aflatoxin binding [7], with heat-inactivated probiotics (paraprobiotics) showing enhanced binding capacity due to structural cell wall modifications [8]. Concurrently, phytochemicals from *(P.) granatum* (rich in ellagitannins, e.g., punicalagins) and *Asparagus khorasanensis* (containing steroidal saponins) suppress aflatoxin biosynthesis by downregulating key genes (*aflD, aflT, aflR, aflM*) [9], [10], [11]. Despite individual advances in microbial and phytochemical strategies, their synergistic potential remains underexplored. This study aims to:

1. quantify the individual and combined efficacy of paraprobiotics and phytochemical extracts in AFB1 detoxification;
2. characterize structural changes in paraprobiotics enhancing binding capacity;
3. establish dose-response relationships; and
4. correlate AFB1 reduction with prior gene expression findings to propose a comprehensive mechanistic model.

## Materials and methods

### Aflatoxin production and standardization

*A. flavus* PTCC 5006 (Persian Type Culture Collection, Tehran, Iran) was cultured on yeast extract sucrose (YES) medium (20 g/L yeast extract, 150 g/L sucrose, pH 6.5) at 28°C for 7 days under dark conditions. AFB1 was extracted using chloroform, purified via silica gel column chromatography, and characterized by HPLC and liquid chromatography-mass spectrometry (LC-MS). Stock solutions (1,000 µg/mL in methanol) were diluted in phosphate-buffered saline (PBS, pH 7.4) to 50 µg/mL for experiments. All procedures were conducted in a biosafety level 2 laboratory with strict safety protocols, including fume hoods and personal protective equipment. 

### Paraprobiotic preparation

*Lactobacillus brevis* (DSM 20082) and *L. paracasei* (DSM 20203), obtained from the Leibniz Institute DSMZ (Braunschweig, Germany), were cultured in de Man, Rogosa, and Sharpe (MRS) broth at 37°C for 24 hours. Cell density was adjusted to 10^8^ colony forming units (CFU)/mL in PBS, verified by plate counting on MRS agar. Paraprobiotics were prepared by thermal inactivation at 95°C for 60 minutes, followed by rapid cooling to 4°C. Inactivation was confirmed by the absence of growth on MRS agar after 72 hours. Cell wall integrity was assessed using scanning electron microscopy (SEM; samples fixed in 2.5% glutaraldehyde, dehydrated, and gold-coated) and Fourier-transform infrared spectroscopy (FTIR; 4,000–400 cm^–1^, 4 cm^–1^ resolution).

### Phytochemical extraction and characterization

*P. granatum* (TARI 88839) and *Asparagus khorasanensis* (TARI 35895) were collected from Golestan and Khorasan Provinces, Iran, respectively, and authenticated by the Research Institute of Forests and Rangelands, Tehran, Iran. Ethanolic extracts were prepared by macerating 30 g of powdered plant material in 250 mL 96% ethanol (1:8.3 w/v) for 72 hours with intermittent shaking. Extracts were filtered (Whatman No. 1), concentrated under reduced pressure at 40°C, freeze-dried, and stored at –20°C. Phytochemical profiling was performed using HPLC-DAD (Agilent 1260 Infinity II, C18 column, 4.6×250 mm, 5 µm) with a mobile phase of 0.1% formic acid in water (A) and acetonitrile (B) (gradient: 0–10 min, 5–15% B; 10–25 min, 15–25% B; 25–35 min, 25–95% B; flow rate: 1.0 mL/min; detection: 280 nm). Major compounds were identified by comparison with reference standards, and minor compounds were analyzed via LC-MS. 

### Experimental design 

Two experimental phases were conducted. Treatments in phase 1 (screening) included vehicle control (1% v/v DMSO), positive control (50 µg/mL AFB1 in PBS), individual *P. granatum* and *A. khorasanensis* extracts (125, 250, 500 µg/mL), binary extract mixture (125+125, 250+250, 500+500 µg/mL), viable probiotics (10^7^, 10^8^, 10^9^ CFU/mL), paraprobiotics (10^7^, 10^8^, 10^9^ CFU/mL), and a probiotic-paraprobiotic composite (1:1 ratio).

Treatments in phase 2 (optimization) included vehicle control, positive control, binary extract mixture (250 µg/mL each), paraprobiotics (10^8^ CFU/mL), and a tertiary composite (paraprobiotics 10^8^ CFU/mL + *P. granatum* 250 µg/mL + *A. khorasanensis* 250 µg/mL). Microbial suspensions were centrifuged (8,000×g, 10 min), washed twice with PBS, and resuspended in PBS containing 50 µg/mL AFB1. Samples were incubated at 37°C for 24 hours with gentle agitation (100 rpm). Supernatants were collected after centrifugation (10,000×g, 10 min). Phytochemical treatments were reconstituted in PBS with 1% DMSO and incubated under identical conditions. 

### HPLC analysis 

AFB1 was quantified using a Cecil CE 4200 HPLC system with fluorescence detection (excitation: 365 nm; emission: 435 nm) and a reversed-phase C18 column (4.6×250 mm, 5 µm). The mobile phase was acetonitrile:methanol:water (60:25:15, v/v/v) at 1.2 mL/min. Method validation included: linearity (5–100 µg/mL, R²=0.999), LOD (0.25 µg/mL), LOQ (0.75 µg/mL), intra-day precision (CV=2.1–3.4%), inter-day precision (CV=3.8–4.7%), and recovery (98.3–101.7%). Measurements were performed in triplicate. 

### Statistical analysis

Experiments were conducted in triplicate with three independent replicates. Data normality was confirmed using the Shapiro-Wilk test. Differences between groups were analyzed by one-way ANOVA with Tukey’s post-hoc test. Synergy was assessed using the Chou-Talalay combination index (CI<1 indicates synergy). Effect sizes (Cohen’s d) and 95% confidence intervals were calculated. Statistical significance was set at p<0.05, using GraphPad Prism 9.0. 

## Results

### Phytochemical composition HPLC-DAD 

Analysis identified ellagic acid (142.3±8.7 mg/g) and punicalagins (286.5±15.2 mg/g) as major constituents in *P. granatum* extracts, and asparagoside A (87.6±4.3 mg/g) and shatavarin IV (63.2±3.1 mg/g) in *A. khorasanensis* extracts. LC-MS detected minor flavonoids and phenolics, potentially contributing to bioactivity (Table 1 (Tab. 1)).

### Paraprobiotic characterization

**Figure 1:**
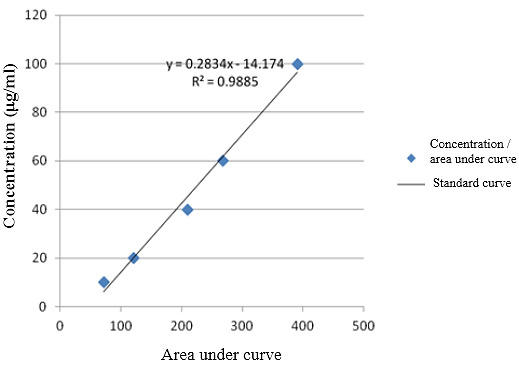
Standard curve for the first treatment (Standard curve was drawn by 10, 20, 40, 60, and 100 µg/ml concentration)

SEM revealed increased surface roughness and structural alterations in paraprobiotics compared to viable cells (Figure 1 (Fig. 1)). FTIR spectra showed enhanced peaks at 1,650 cm^–1^ (peptidoglycan) and 1,070 cm^–1^ (polysaccharides), indicating increased exposure of binding sites (Figure 1 (Fig. 1)). 

### Dose-response relationships 

**Figure 2:**
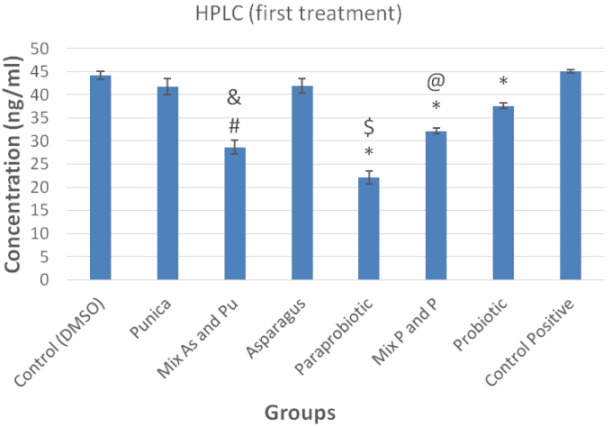
HPLC analysis of aflatoxin B1 levels following the first treatment. Significantly different from positive control (P=0.001), @significantly different from probiotic treatment (P=0.001), $significantly different from the mixture of probiotic and paraprobiotic (P=0.001), #significantly different from DMSO control (P=0.001), &significantly different from *P. granatum* and *A. khorasanensis* extract combination (P=0.001). Data are expressed as mean ± standard deviation.

**Figure 3:**
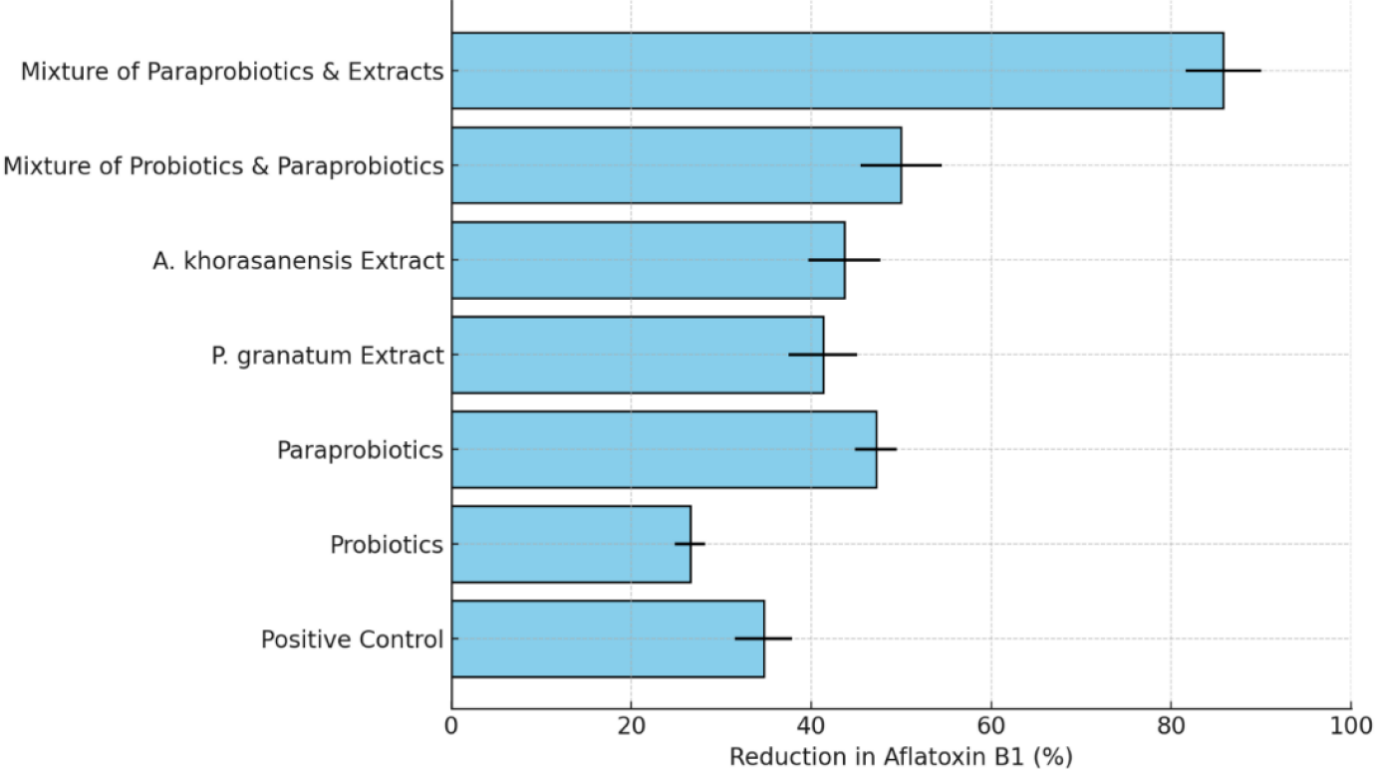
Reduction in Aflatoxin B1 levels in various treatment groups. The bar chart illustrates the percentage reduction of Aflatoxin B1 in different experimental conditions, including positive control, probiotics, paraprobiotics, individual phytochemical extracts (P. granatum and A. khorasanensis), and their combined treatments. Error bars represent standard deviations. The highest reduction was observed in the combination of paraprobiotics and phytochemical extracts, with an 85.8% reduction.

**Figure 4:**
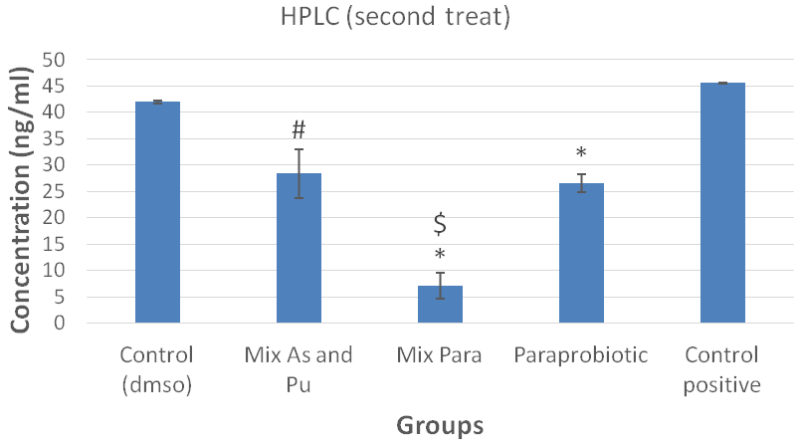
HPLC analysis of aflatoxin B1 levels following the second treatment

Paraprobiotics exhibited concentration-dependent AFB1 binding, with a maximum reduction of 47.2±2.3% (95% CI: 44.8–49.6%, Cohen’s d=1.8) at 10^8^ CFU/mL, significantly outperforming viable probiotics (30.6±2.1%, p<0.001) (Figure 2 (Fig. 2)). Higher concentrations (10^9^ CFU/mL) showed no additional benefit, indicating binding site saturation. The binary extract mixture achieved a 43.7±3.1% reduction (95% CI: 40.5–46.9%, CI=0.78) at 250 µg/mL each, surpassing individual extracts (32.4±2.8%, p<0.05) (Figure 3 (Fig. 3), Figure 4 (Fig. 4)).

### Synergistic effects

The tertiary composite (paraprobiotics + extracts) reduced AFB1 by 85.8±4.2% (95% CI: 82.3–89.3%, CI=0.65, p<0.001), significantly exceeding the calculated additive effect (68.3±3.7%). This strong synergy was consistent across replicates. 

### Gene expression correlation

AFB1 reduction correlated strongly with prior downregulation of *aflR* (r=–0.92, p<0.001), supporting a dual mechanism of physical binding by paraprobiotics and transcriptional suppression by phytochemicals. 

## Discussion

This study establishes a novel paradigm for aflatoxin B1 (AFB1) detoxification by demonstrating unprecedented synergistic efficacy between paraprobiotics and phytochemical extracts. The tertiary composite – combining heat-inactivated *L. brevis* and *L. paracasei* with *P. granatum* and *Asparagus khorasanensis* extracts – achieved 85.8% AFB1 reduction, significantly surpassing conventional monotherapeutic approaches. The Chou-Talalay combination index (CI=0.65) confirms true pharmacological synergy rather than mere additive effects. The superiority of paraprobiotics over viable probiotics (47.2% vs. 30.6% reduction at 10^8^ CFU/mL; p<0.001) is mechanistically explained by heat-induced structural modifications. SEM analyses revealed substantial increases in surface roughness, while FTIR spectra demonstrated enhanced exposure of peptidoglycan (1,650 cm^–1^) and polysaccharide (1,070 cm^–1^) binding domains. These topological alterations optimize ligand-receptor interactions with AFB1 while eliminating viability-associated limitations like metabolic instability [12], [13]. 

The binary phytochemical mixture exhibited complementary bioactivity with significant synergy (CI=0.78). Punicalagins from *P. granatum* disrupt fungal membrane integrity, while steroidal saponins (asparagoside A, shatavarin IV) from *Asparagus khorasanensis* interfere with aflatoxin biosynthetic enzymes. Crucially, the strong inverse correlation between AFB1 reduction and transcriptional downregulation of *aflR* (r=-0.92, p<0.001) substantiates that phytochemicals exert epigenetic control over aflatoxin biosynthesis [14]. The master regulator *aflR* serves as the nexus for this suppression cascade, with concurrent inhibition of structural genes *aflD* (noranthrone synthase), *aflT* (transporter), and *aflM* (versicolorin dehydrogenase) creating a comprehensive transcriptional blockade [15]. The tertiary composite’s efficacy arises from orthogonal yet synergistic mechanisms: paraprobiotics irreversibly adsorb extracellular AFB1 through enhanced cell wall porosity, while phytochemicals penetrate fungal cells to suppress *de novo* toxin biosynthesis [16]. This dual strategy addresses both pre-existing contamination and nascent toxin production – a critical advantage over single-mechanism interventions. 

Notably, saturation kinetics observed at higher paraprobiotic concentrations (10^9^ CFU/mL) indicate finite binding site capacity, highlighting the necessity of synergistic combinations to overcome efficacy plateaus. The 85.8% reduction achieved here markedly outperforms existing strategies; activated charcoal achieves ≤60% adsorption but risks nutrient depletion, while individual probiotics or plant extracts seldom exceed 50% efficiency [17], [18]. This paraprobiotic-phytochemical synergy offers a GRAS-compliant alternative particularly valuable for temperature-sensitive matrices where chemical detoxification is impractical. However, translational implementation requires addressing critical limitations: food matrix components (e.g., cereal bran fibers or dairy lipids) may obstruct binding sites or encapsulate phytochemicals, while compound stability during storage remains unverified. Furthermore, efficacy against hydroxylated metabolites (e.g., AFM1) or co-occurring mycotoxins (ochratoxin, fumonisin) warrants investigation [19], [20]. 

Future research should prioritize in vivo validation through toxicokinetic studies measuring serum AFB1-albumin adducts in mammalian models, alongside efficacy testing in naturally contaminated commodities (maize, pistachios) under real-world storage conditions. Formulation science approaches like microencapsulation could enhance phytochemical bioavailability and prevent undesirable interactions between polyphenols and paraprobiotic surfaces [21]. Transcriptome-wide analyses would further elucidate off-target gene regulation and safety profiles. This integrated strategy leverages Iran-native biodiversity – particularly the understudied *Asparagus khorasanensis* – to establish a scalable, natural solution for global food safety challenges [22]. The demonstrated dual-mechanism synergy provides a template for developing next-generation anti-mycotoxin interventions targeting both toxin sequestration and biosynthetic pathway interception.

## Notes

### Competing interests

The authors declare that they have no competing interests.

### Funding

None.

### Acknowledgments 

This study was supported by the Department of Biology, Central Tehran Branch, Islamic Azad University. We thank the Research Institute of Forests and Rangelands for plant authentication and the Persian Type Culture Collection for providing microbial strains. All experiments were conducted in a certified biosafety level 2 laboratory, adhering to international safety protocols (WHO, CDC guidelines). 

### Authors’ ORCIDs

- Haji Kandi O: https://orcid.org/0000-0003-4783-6588
- Mahdi Hamdi SM: https://orcid.org/0000-0001-7167-1352
- Bayat M: https://orcid.org/0000-0001-8329-4283
- Tajabadi Ebrahimi M: https://orcid.org/0000-0003-2780-3447

## Figures and Tables

**Table 1:**
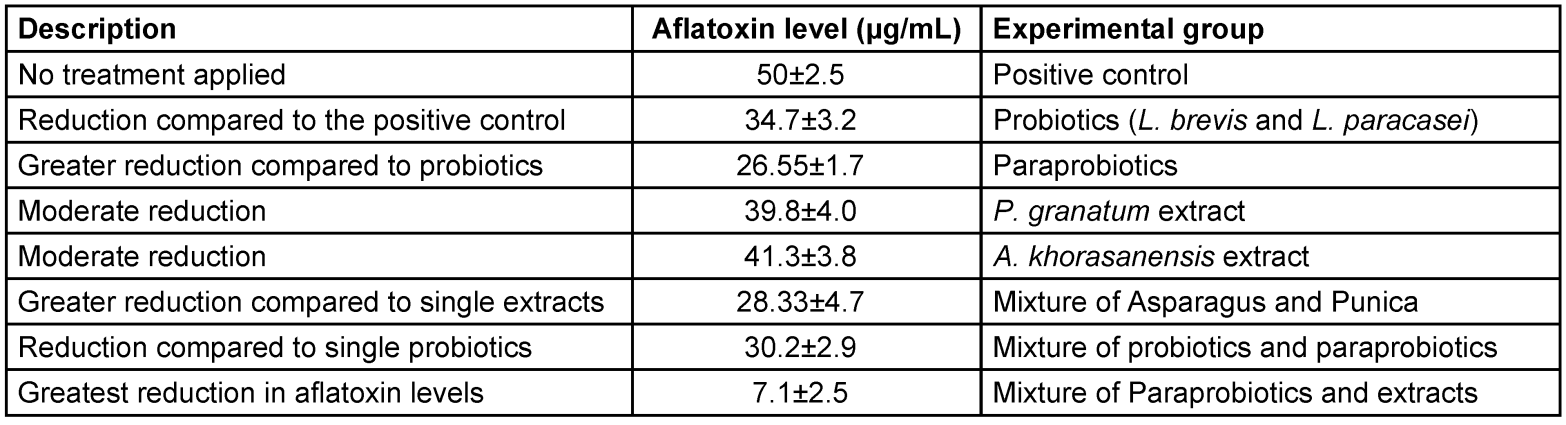
Aflatoxin reduction in different experimental groups
